# Low-Cost Flexible ZnO Microwires Array Ultraviolet Photodetector Embedded in PAVL Substrate

**DOI:** 10.1186/s11671-018-2701-4

**Published:** 2018-09-10

**Authors:** Xinyu Sun, Fahad Azad, Shuangpeng Wang, Lingzhi Zhao, Shichen Su

**Affiliations:** 1 Institute of Optoelectronic Material and Technology, South China Normal University, Guangzhou, 510631 People’s Republic of China; 2 School of Natural Sciences (SNS), National University of Science and Technology (NUST), H-12, Islamabad, Pakistan; 3 Institute of Applied Physics and Materials Engineering, University of Macau, Macau, 999078 People’s Republic of China; 4 Guangdong Provincial Engineering Technology Research Center for Low Carbon and Advanced Energy Materials, Guangzhou, 510631 People’s Republic of China

**Keywords:** ZnO microwire array, Ultraviolet photodetector

## Abstract

With the advent of wearable device technology, fabrication of inorganic semiconductor devices on flexible organic substrates is of great interest. In this paper, a fascinating method and a low-cost flexible substrate material polyvinyl alcohol (PVAL) have been utilized to embed ZnO microwire (MW) array to produce ultraviolet (UV) photodetector (PD) with decent photoresponsivity. The flexible PVAL substrate is relatively cheap and has better bendability as compared to polyethylene terephthalate (PET) and other traditional flexible substrate materials, which makes it unique in comparison to traditional devices. The device shows a current photoresponsivity of 29.6 A/W in the UV spectral range (350 to 380 nm) and maintains an excellent detection performance with even a bending angle of 180°. In dark, a low current of 1.4 μA at 5 V bias and response time of 4.27 ms was observed. In addition to the excellent device performance at wide bending angles, the fabricated device also performs well with the bending radii close to 0. Therefore, ZnO MW array PD has a great potential for the real-time monitoring of harmful UV exposure to warn the users for the appropriate arrangement avoidance.

## Background

The detection of ultraviolet (UV) light is important in fields like astronomy, environmental monitoring, and in many biological processes [1]. UV light exposure causes mutation in p53 tumor suppressor genes that causes skin cancer [2]. Therefore, to prevent the injurious effects of sunlight on human health, proper monitoring of these radiations is essential. Furthermore, it is more appropriate to monitor individual’s UV exposure as the amount of sun exposure varies from person to person [3]. With the advent of wearable technology, users can now monitor the UV exposure in real time and they can also receive alerts regarding surrounding radiation conditions and their biometric parameters [4]. Therefore, a wearable device with flexible UV PD that could render efficient detection under the bending conditions (required for performing daily activities of user) is essential to monitor individual’s UV exposure.

ZnO, a typical II–IV direct-gap semiconductor, has a wide bandgap (3.37 eV at 300 K) and large excitation binding energy (60 meV). It is one of the most compatible materials for photonic applications such as UV photodetectors and light-emitting diodes (LEDs) [1, 5]. The dominant crystalline structure of ZnO is hexagonal wurtzite with spontaneous polar angle along the c-axis, which has been observed in various ZnO nanostructures such as thin film [6, 7], nanorods [8, 9], nanowires [10, 11], nano tetrapods [12, 13], nanobelts [14, 15], and nanocombs [16, 17]. Patterning and alignment of these nanostructures is crucial for device fabrication [18]. To align nanorods and nanowires, several methods such as horizontal manual alignment [19, 20], dielectrophoresis [21, 22], and self-alignment [23] have been explored. Regardless of distinctive properties of these nanostructures, large scale production of these devices is limited due to one by one manufacturing process. The growth of ZnO films using cheap and simple methods has attracted the interest of many researchers [24]. Usually, nanostructures of ZnO are fabricated by both chemical and physical vapor deposition methods. Many advanced techniques such as chemical vapor deposition (CVD) [25], metal organic chemical vapor deposition (MOCVD) [26, 27], pulsed laser deposition (PLD) [28, 29], radio-frequency magnetron sputtering (RFMS) [30, 31], and electron beam evaporation (EBE) [32, 33] have been utilized to grow ZnO films. Wet chemical method such as Sol-gel deposition has also been utilized with several casting methods such as dip [34], spin [35], and spray coating to grow ZnO. Sol-gel is an inexpensive and simple method for large scale and roll to roll production. All the discussed method provides ZnO films with large surface area which needs further patterning to meet design requirement of the device. For the patterning of these devices, slow process like photolithography is used [36]. Moreover, etching constituents which are used for patterning are not compatible with the flexible substrate in some cases [37].

Other manufacturing approaches have also been utilized to prepare on-demand ZnO pattern shapes. Some novel approaches have proven to be limited in terms of device cost and performance [26, 32]. Polycrystalline ZnO with a huge amount of grain boundaries fabricated by electrospinning was found to effectively reduce dark current and significantly increase photoresponsivity. Generally, there are two types of PD: photovoltaic PD and none-junction/metal-semiconductor-metal (MSM) PD [19]. Photovoltaic PD has two types: Schottky and P-N/PIN junction [38], whereas MSM PD has a much simpler structure and fabrication process as compared to photovoltaic PD. Therefore, MSM PDs are preferable in practical applications and it is worthwhile to investigate the factors which improve the performance of these detectors [39].

The selection on flexible substrate of ZnO UV PD is crucial to device performance too. According to the variety of nanostructures, shapes and sizes, and synthesis methods, ZnO has been synthesized on diverse substrates in the literatures. Polyethylene terephthalate (PET) and poly urethane (PU) were frequently utilized for flexible ZnO UV devices. Zhang et al. fabricated a ZnO UV PD based on flexible PU fibers; however, the device has worse performance of low photocurrent attributed to the rough surface of woven PU fibers [40]. In some ZnO nanowire UV PD, ZnO nanowires need to be synthesized directly on the substrate in a furnace with extremely high temperature. But almost every organic flexible substrate cannot endure the high temperature of own to low melting point. As a result, reasonable device structure and selection of flexible substrate material lead to the performance of flexible ZnO UV PD.

In this study, a ZnO MW array embedded in soft PVAL substrate has been demonstrated to be an effective UV PD. We used liquid PVAL glue to cover the most part of ZnO MW array except the surface of hexahedron structure of ZnO MWs. The PVAL glue was then dried, and Au interdigital electrodes were deposited. This PD device has an excellent flexibility and bending strength, which is proven by its ability to work under large bending angles and bending radii for multiple cycles. This PD device was found to have a rapid response time of 4.27 ms and high photoresponsivity of 29.6 A/W. Thus, it is an excellent candidate for wearable devices to monitor UV exposure in order to reduce the possible health hazards.

## Methods/Experimental

**Fig. 1:**
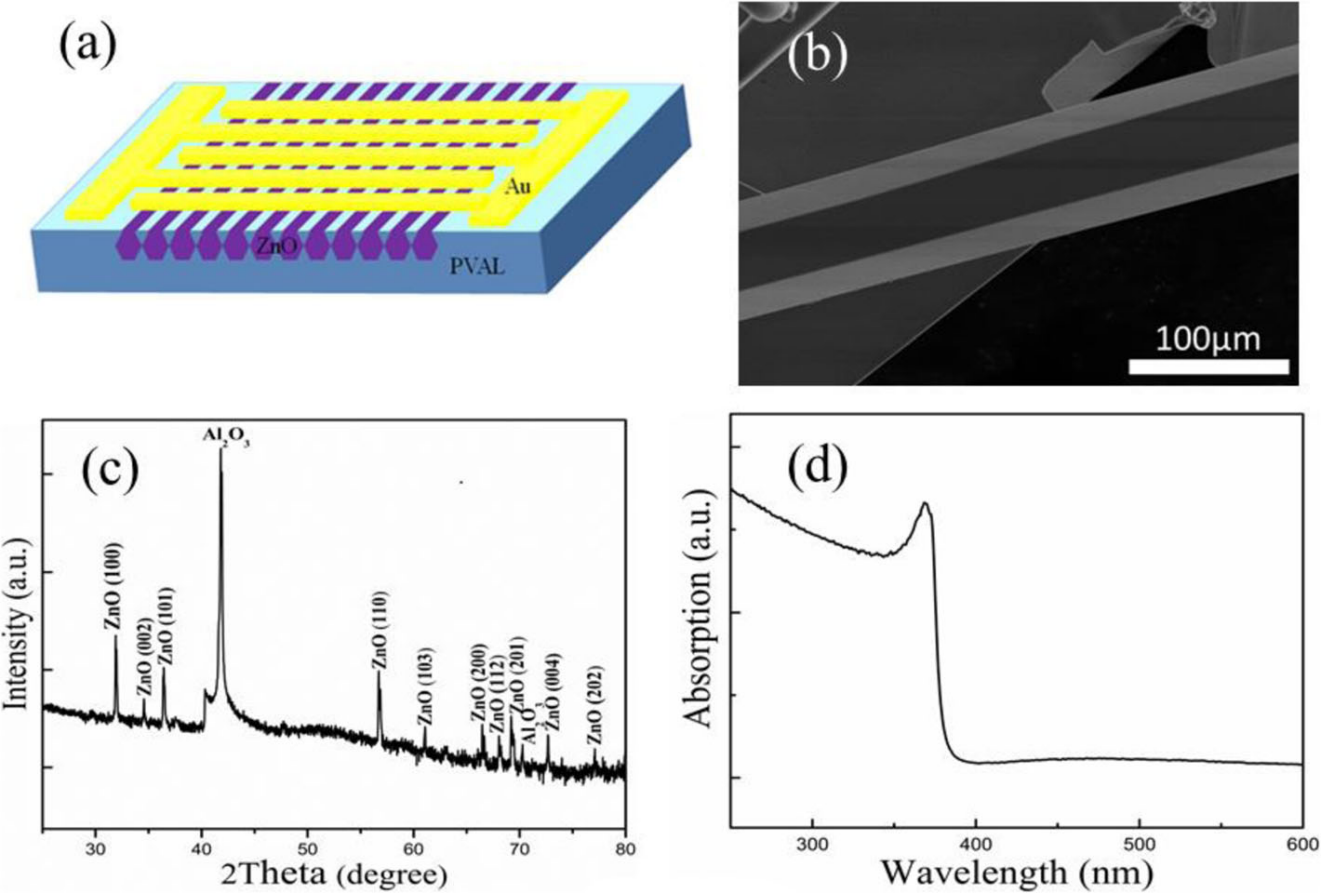
**a** The schematic of ZnO MW array UV PD device. **b** SEM micrograph of the synthesized ZnO MWs. **c** XRD pattern of the ZnO MW sample on the sapphire substrate. **d** Absorption spectrum of the ZnO MWs

**Fig. 2:**
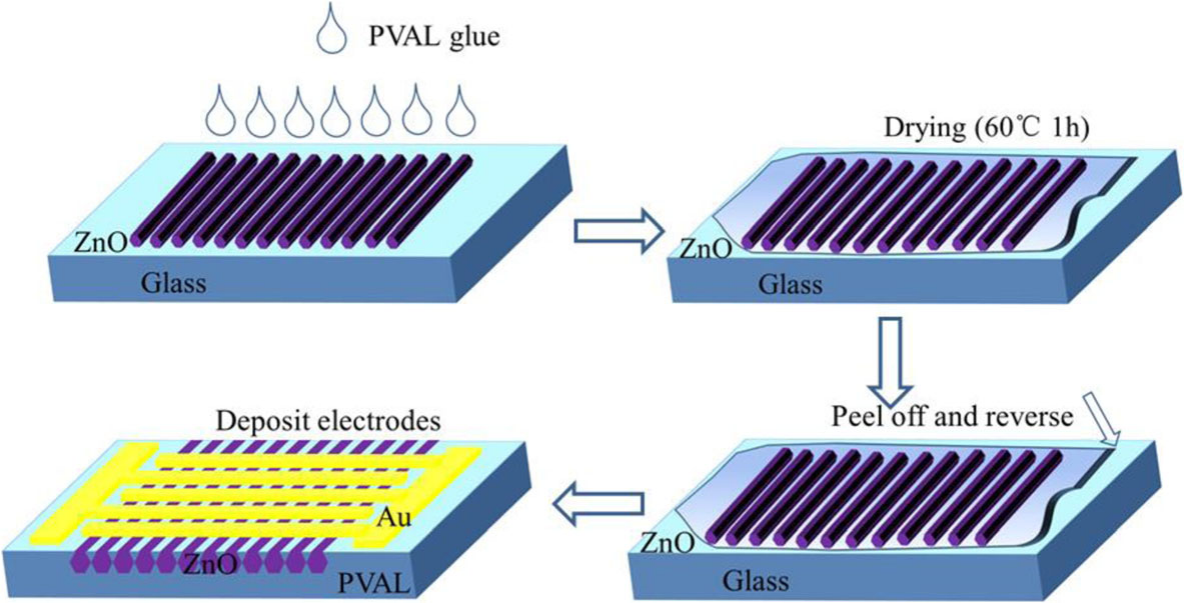
The schematic of fabrication of the photodetector

A schematic of the ZnO MW array UV PD is presented in Fig. 1a. The diameter of MWs is 40–50 μm. The MWs were grown by the chemical vapor deposition (CVD) technique. 99.99% powder of Zn sintered to 980 °C for 1 h and turned to gas of Zn in N_2_ ambient, introduced O_2_ and stayed 980 °C for 1 h, and cooled down to room temperature and got ZnO MWs; more experiment details could be taken in our previous work [41]. ZnO MW arrays of large diameter (40–50 μm) and length (3–5 mm) have been utilized in Fig. 1b for this study. The glass substrate was washed by acetone, ethanol, and deionized water successively. The ZnO MW array was then moved to the glass substrate and compelled to adapt to the surface of glass substrate. PVAL glue was then added dropwise (1 ml) on the ZnO MW arrays evenly. The substrate with ZnO MW array was then kept into drying oven (60 °C) for 1 h. The ZnO MW array structure was then peeled off from the glass substrate. Au interdigital electrodes with five pairs of electrode fingers (the gap between adjacent electrodes is 100 μm, finger length is 200 μm) were then deposited on the ZnO MW arrays and PVAL substrate to complete the device fabrication. Figure 2 could explain the fabrication of this photodetector device briefly. This configuration protects ZnO MW arrays as they are embedded in the flexible PVAL substrate. Only the surface of these MWs was exposed to make contact with Au interdigital electrodes.

The morphology and structure of ZnO MWs was characterized by scanning electron microscope (SEM, ZEISS Gemini 500), optical microscope, and X-ray diffractometer (XRD, BRUKER D8 ADVANCE Germany). The absorption spectrum was obtained using a continuous He−Cd (325 nm) laser as an excitation source. The room temperature electrical and photoresponse properties of the fabricated device were measured with a semiconductor characterization system (Agilent B2901A).

## Results and Discussion

Figure 1b represents a typical SEM image of the synthesized MWs. The MWs were found to have diameters of 40–50 μm and lengths of several millimeters. The XRD pattern of the ZnO MWs in Fig. 1c indicates wurtzite structure; no secondary phase was detected in the XRD pattern [42]. The absorption spectrum of the as-prepared ZnO MWs is depicted in Fig. 1d indicating good crystallinity with low deficiencies [43].

**Fig. 3:**
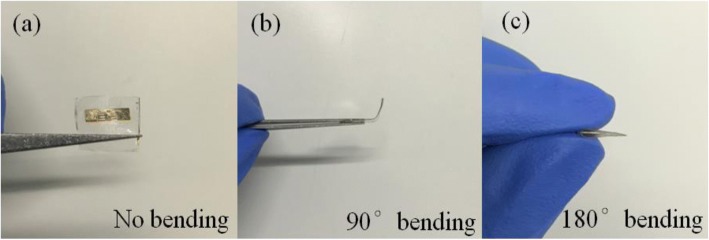
The schematic of ZnO MW array PD when there is **a** no bending, **b** 90° bending, and **c** 180° bending

**Fig. 4:**
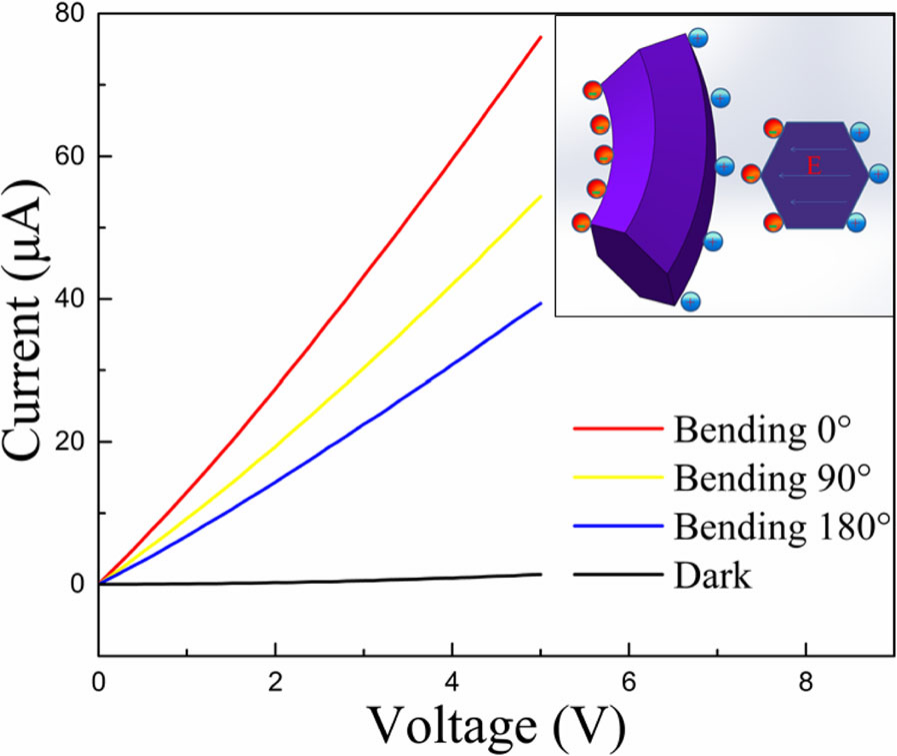
I–V characteristic in dark and under UV illumination at different bending angles. The inset (left) shows the bending strain-induced nonmobile ionic charges at the outer (positive) and inner (negative) surfaces of the ZnO MW, and the inset (right) shows the piezo-induced electric field and piezopotential distribution at the cross-section of the bent ZnO MW

Figure 3 shows fabricated ZnO MW array PD with no bending (Fig. 3a), 90° bending (Fig. 3b), and 180° bending (Fig. 3c). Figure 4 shows I–V characteristics of ZnO MW array PD devices with and without UV light illumination, 90° bending, and 180° bending. The linear behavior indicates ohmic contact due to lower work function of ZnO (4.5 eV) compared to that of Au (5.1 eV) [44], thus leading to the band distortion and the formation of the depletion region adjacent to the interface. Once the junction is illuminated by the UV light (380 nm), the electrons and holes generated within the depletion region, immediately move in opposite directions by the built-in potential that gives rise to generation of circuit current. The current increased dramatically, which illustrates that the flexible PD possesses high sensitivity. The flexible UV PDs usually have lower photocurrent compared to the traditional PDs based on Si/SiO_2_ substrate due to the poor contact between the material and flexible substrate. But in this study, the embedded ZnO MW arrays have excellent contact with PAVL substrate which is shown from the high sensitivity. The Fermi energy level in ZnO is higher than that of Au. Therefore, electrons will diffuse from ZnO side to Au and a potential barrier will be established that will oppose the further electron flow across the Schottky junction. When an external strain is applied, it creates a negative piezopotential at the interface of Schottkey junction which forces electrons to move away from the interface. The repulsion of electrons from the interface will further deplete the interface and increase the height of potential barrier. Although increase in barrier height and width is suitable for the photoexcited extraction and separation, it will alter the transport behavior due to piezoresistance effect. However, the change in the transport behavior is a symmetrical effect which only alters the resistivity of the semiconductor not the interface properties. In this work, the charge transport process due to unsymmetrical variation of current under negative and positive bias is dominated by piezoelectric effect. Hence, the photocurrent decreases with the increase in bending angle.

Wang et al. has discussed the effect of piezoelectric effect on electronic transport properties of ZnO nanowires (NWs) [45]. The positive and negative charging of outer stretched (positively strained) and inner compressed (negatively strained) surface, respectively, in a bent ZnO NW were assigned to be the reason of change in IV characteristics (Fig. 4 inset). The induction of these static ionic charges happens due to the piezoelectric effect. The piezoelectric field along the NW is given by *E* = *ɛ*/*d*, where *ɛ* and *d* are strain and piezoelectric coefficient, respectively. Two mechanisms were postulated to describe reduction in conductance of NW: (i) the effective carrier density of ZnO NW lowers as the free electrons trap at the inner arc and ions on outer arc surface of the bent NW; (ii) the reduction in the width of conducting channel due to repulsion of electrons across the width, by the induced piezoelectric field.

In our work, the soft layer of PVAL in this MW array UV PD device plays a crucial role in electronic transport. The electron trapping at the interface states builds up depletion region inside the MWs which results in decreasing the effective channel area and creating the surface potential barrier ɸ_s_ between the MWs and the PVAL dielectrics. When ZnO MW UV PD devices are bent, the electron trapping at the interface states is influenced by different charged surfaces induced by the piezoelectric effect, resulting in the change of transport characteristics.

In the unbent ZnO MW UV PD, the trapping of electrons produces ɸ_s_ and the band bends upward. When external force is applied to bend the ZnO MW array PD, the applied strain also bends the ZnO MWs. The bending of MWs induces piezoelectric potential ɸ_PZ_ due to movement of Zn^2+^ ions away from O^2−^ ions. The effective potential at the interface varies due to the effect of ɸ_PZ_ on ɸ_s_ by altering electronic transport properties of ZnO MW array PD due to variation in electron trapping. Negative charge appears on the compressed side of ZnO MW which reduces electron trapping due to repulsion on this side. Whereas, stretched ZnO MW side has positive charge which enhances the trapping of free electrons.

**Fig. 5:**
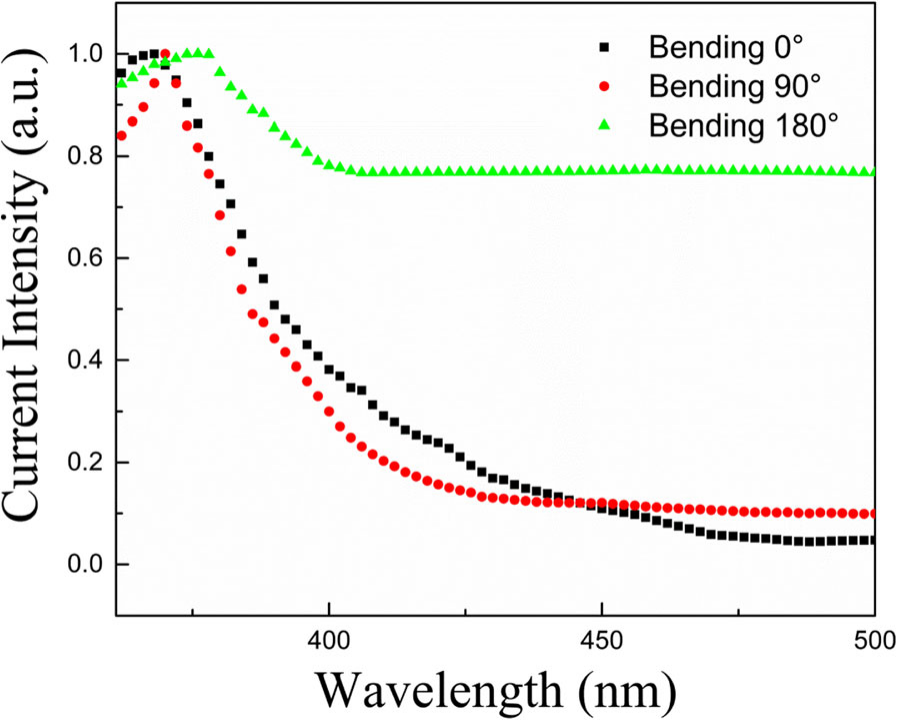
The photoresponse wavelength of ZnO MW array PD at different bending angles (0°, 90°, and 180°)

A red shift in the photoresponse wavelength (Fig. 5) was observed by decreasing the bending angle. First principle DFT simulations have been performed on this ZnO MWs under pure tensile and compressive strains to evaluate strain-induced change in the bandgap [46]. For these simulations, the ZnO MWs were strained axially. All the structural optimizations and energy calculations were performed based on pseudopotentials with localized atomic orbital basis sets within the Perdew-Burke-Ernzerhof general gradient approximation implemented in the code SIESTA [47, 48].

**Fig. 6:**
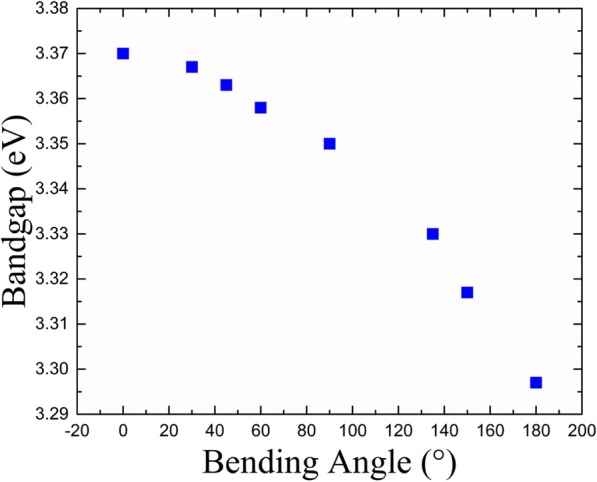
Bandgap of ZnO MWs at different bending angles

To obtain a relationship between the bending angle and bandgap, bandgaps at different bending angles were measured; the data is shown in Fig. 6. The bandgap can also be calculated as a function in the framework of a six-band effective-mass envelope function theory [49]. A significant reduction in the bandgap was observed with decrease in the bending angles. The bandgap decreases from 3.37 eV (bulk) to 3.29 eV due to increase in bending angle from 0° to 180°, respectively, which is in agreement with the six-band effective-mass envelope function theory.

**Fig. 7:**
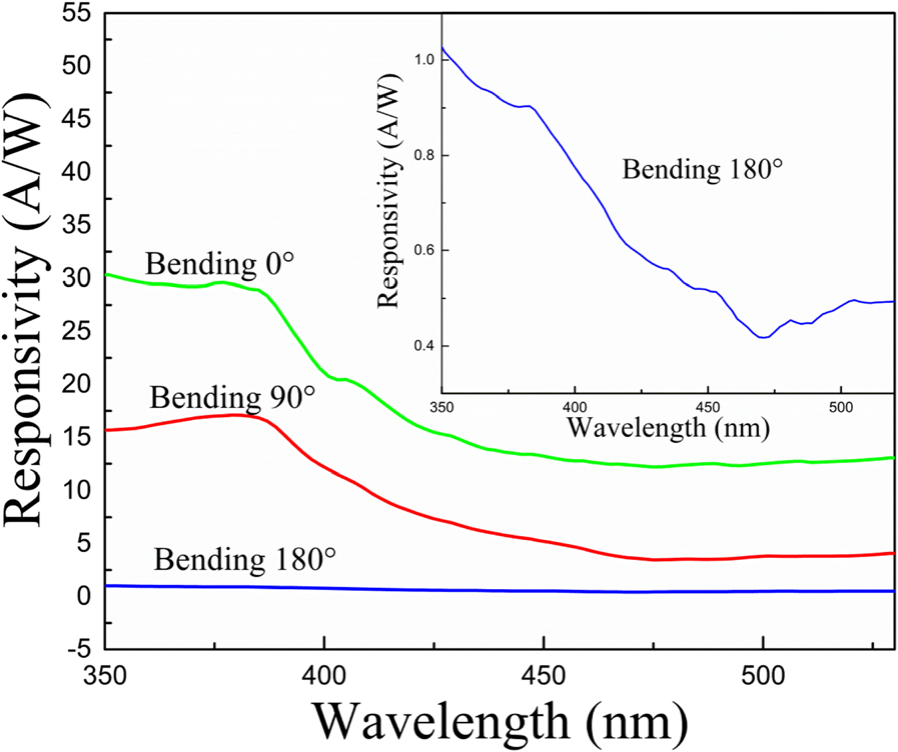
Spectral dependence of the photoresponsivity of ZnO MW array UV PD at incident power of 1 μW with 5 V bias at different bending angles (0°, 90°, and 180°)

The bandgap and resistance of these ZnO MWs were changed with bending along with photocurrent and the photoresponsivity of the ZnO MW array UV PD also changed. Figure 7 shows the spectral photoresponsivity of ZnO MW array UV PD at different bending angles. It is apparent that the photoresponsivity decreases with the increase in bending angles. The photoresponsivities were measured to be 29.6A/W, 17.1A/W, and 0.95A/W for the bending angle of 0°, 90°, and 180°, respectively. Although, the external stress reduces the photoresponsivity of ZnO MW array UV PD, but even at bending angle of 180°, it is still responsive to UV radiations. Furthermore, the photoresponsivity of ZnO MW array UV PD device were recovered on unbending the device.

**Fig. 8:**
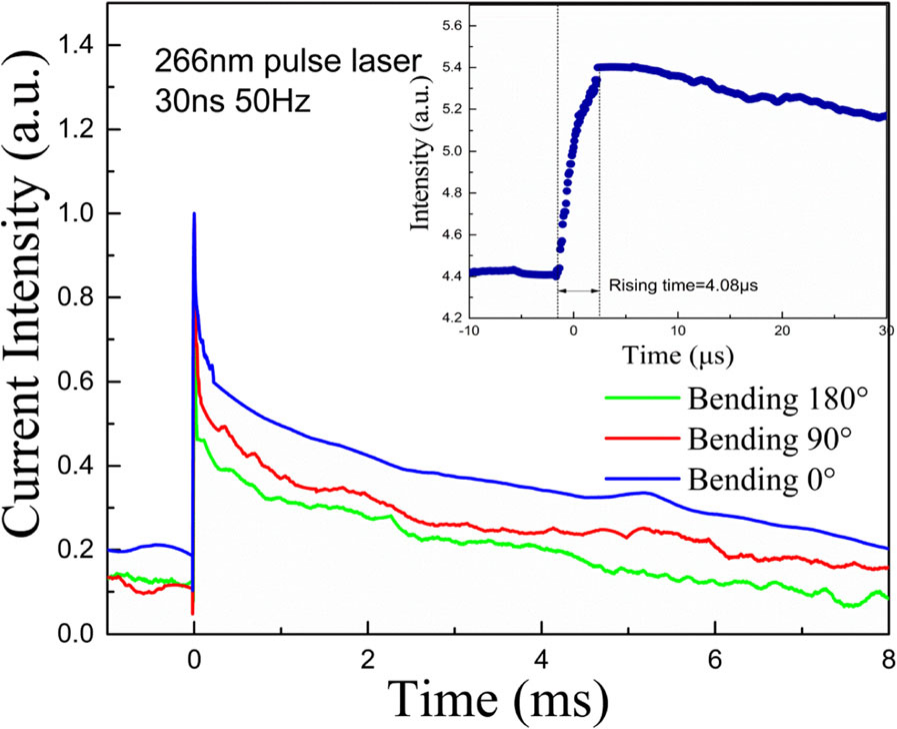
The responsive time of ZnO MW array UV PD device for 266 nm pulsed laser illumination for 30 ns at a frequency of 50 Hz under a bias of 10 V at different bending angles (0°, 90°, and 180°)

**Fig. 9:**
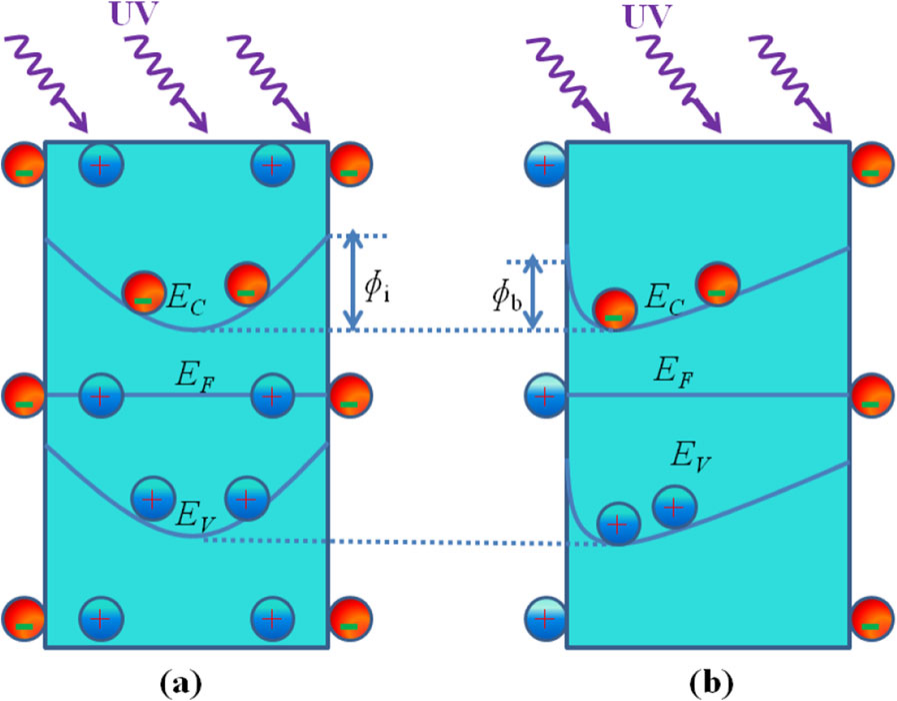
**a** The band diagram for an unbent MW: the conduction and valence band edges are bent near the surface due to the surface pinning of Fermi level. The intrinsic surface recombination barrier *ɸ*_i_ is also shown. **b** The case for a bent MW: the piezo-induced electric field lowers the surface recombination barrier from *ɸ*_i_ to *ɸ*_b_

Figure 8 presents the dependence of decay times on bending angle for the ZnO MWs array PD device. 266 nm Nd:YAG pulsed laser was used to illuminate the PD device for 30 ns (pulse width) and a bias of 10 V was applied. A reduction in the decay time with increase in the bending angle was noticed. The corresponding values for decay time was found to be 6.18 ms, 6.02 ms, and 4.27 ms for the bending angles of 0°, 90°, and 180°, respectively. The rising time was found to be 4.08 μs which is limited by the pulse width (inset in Fig. 8). The reduction in the decay time can be explained by considering the band diagrams of these MWs for unbent and bent cases. A space-charge depletion layer exists at the surface of these n-type ZnO MWs, and fermi energy level pins between the forbidden gap at the surface [50, 51]. The depletion layer width depends upon MW’s thickness and its atmosphere and doping level which can be controlled by manipulating these factors. In the unbent ZnO MW, conduction band edge (*E*_c_) and valence band edge (*E*_v_) bend upward near the surface of MW and the space-charge depletion region extends up to *E*_c_ and *E*_v_ band, as shown schematically in Fig. 9. Therefore, photoexcited holes migrate to the surface and electron prefers to stay in the inner part of the MW. The high surface to volume ratio of MWs plays an important role in easy trapping of holes at the surface. The trapping of carriers in surface traps is the dominant recombination mechanism [52]. The separation between photoexcited electrons and holes reduces the recombination of non-equilibrium carriers. Therefore, to recombine with holes at the surface, electrons have to cross a potential barrier ɸ_i_ (Fig. 9a). When surface recombination controls decay time of the persistent photocurrent, the recombination rate is given by exp(−ɸ_i_/*kT*) [52].

For the bending case, the induced piezoelectric field modifies energy bands. At the negatively charged surface of the MW, *E*_v_ moves towards while *E*_c_ moves away from Fermi level. Whereas, near the positively charged surface, both *E*_v_ and *E*_c_ moves closer to Fermi level, as shown in Fig. 9b. The intrinsic recombination barrier ɸ_i_ (Fig. 9a) for the unbent case is higher than that of the potential barrier ɸ_b_ for the bending case (Fig. 9b). Therefore, the recombination rate increases due to reduction in the ɸ_b_ upon bending. The decay times for the bending case also get shorter as it depend upon the recombination barrier.

## Conclusions

In this work, the fabrication of ZnO MW array flexible UV PD embedded in PVAL soft substrate was demonstrated. The process is easy and inexpensive. Good ohmic contacts were created between the Au electrodes and embedded ZnO MW array. The highest response time was found to be 4.27 ms and photoresponsivity to be 29.6 A/W for the fabricated device. Degradation of the device was observed under large bending angles and bending radii, but the UV detection performance was not affected significantly. The effect of bending radii on the performance of the device was also studied. The results suggest that the device is compatible for wearable in situ monitoring UV PDs. This process also shows potential for other devices that need flexibility, such as small-size transistors and solar cells for wearable devices. In addition, the simplicity of the fabrication process might support the idea for custom-made devices or in situ fabrication.

## Abbreviations

CVD: Chemical vapor deposition
EBE: Electron beam evaporation
MOCVD: Metal organic chemical vapor deposition
MSM: Metal-semiconductor-metal
MWs: Microwires
PD: Photodetector
PET: Polyethylene terephthalate
PLD: Pulsed laser deposition
PVAl: Polyvinyl alcohol
RFMS: Radio-frequency magnetron sputtering
UV: Ultraviolet

## References

[1] Monroy E, Omnès F, Calle F (2003). Wide-bandgap semiconductor ultraviolet photodetectors. Semicond Sci Technol.

[2] Nakazawa H, English D, Randell PL, Nakazawa K, Martel N, Armstrong BK, Yamasaki H (1994). UV and skin cancer: specific p53 gene mutation in normal skin as a biologically relevant exposure measurement. Proc Nat Acad Sci.

[3] Kimlin MG, Parisi AV, Wong J (1998) Quantification of personal solar UV exposure of outdoor workers, indoor workers and adolescents at two locations in Southeast Queensland. Photodermatolo. Photo 14 (1): 7–11 10.1111/j.1600-0781.1998.tb00002.x 9582080

[4] Binkley PF (2003). Predicting the potential of wearable technology. IEEE Eng Med Biol.

[5] Özgür Ü, Alivov YI, Liu C, Teke A, Reshchikov M, Doğan S, Avrutin V, Cho SJ, Morkoc H (2005). A comprehensive review of ZnO materials and devices. J Appl Phys.

[6] Gardeniers JG, Rittersma Z, Burger G (1998). Preferred orientation and piezoelectricity in sputtered ZnO films. J Appl Phys.

[7] Petrov I, Orlinov V, Misiuk A (1984). Highly oriented ZnO films obtained by dc reactive sputtering of a zinc target. Thin Solid Films.

[8] Tian ZR, Voigt JA, Liu J, Mckenzie B, Mcdermott MJ, Rodriguez MA, Konishi H, Xu H (2003). Complex and oriented ZnO nanostructures. Nat Mater.

[9] Guo M, Diao P, Cai S (2005). Hydrothermal growth of well-aligned ZnO nanorod arrays: dependence of morphology and alignment ordering upon preparing conditions. J Solid State Chem.

[10] Huang MH, Mao S, Feick H, Yan H, Wu Y, Kind H, Weber E, Russo R, Yang P (2001). Room-temperature ultraviolet nanowire nanolasers. Science.

[11] Huang MH, Wu Y, Feick H, Tran N, Weber E, Yang P (2001). Catalytic growth of zinc oxide nanowires by vapor transport. Adv Mater.

[12] Gedamu D, Paulowicz I, Kaps S, Lupan O, Wille S, Haidarschin G, Mishra YK, Adelung R (2014). Rapid fabrication technique for interpenetrated ZnO nanotetrapod networks for fast UV sensors. Adv Mater.

[13] Yu W, Li X, Gao X (2004). Self-catalytic synthesis and photoluminescence of ZnO nanostructures on ZnO nanocrystal substrates. Appl Phys Lett.

[14] Yan H, He RR, Pham J, Yang P (2003). Morphogenesis of one-dimensional ZnO nano-and microcrystals. Adv Mater.

[15] Pan ZW, Dai ZR, Wang ZL (2001). Nanobelts of semiconducting oxides. Science.

[16] Lao CS, Gao PX, Yang RS, Zhang Y, Dai Y, Wang ZL (2006). Formation of double-side teethed nanocombs of ZnO and self-catalysis of Zn-terminated polar surface. Chem Phys Lett.

[17] Xu F, Yu K, Li G, Li Q, Zhu Z (2006). Synthesis and field emission of four kinds of ZnO nanostructures: nanosleeve-fishes, radial nanowire arrays, nanocombs and nanoflowers. Nanotechnology.

[18] Xu S, Wei Y, Kirkham M, Liu J, Mai W, Davidovic D, Snyder RL, Wang ZL (2008). Patterned growth of vertically aligned ZnO nanowire arrays on inorganic substrates at low temperature without catalyst. J Am Chem Soc.

[19] Soci C, Zhang A, Xiang B, Dayeh S, Aplin D, Park J, Bao X, Lo Y, Wang D (2007). ZnO nanowire UV photodetectors with high internal gain. Nano Lett.

[20] Das SN, Kar JP, Choi JH, Lee TI, Moon KJ, Myoung JM (2010). Fabrication and characterization of ZnO single nanowire-based hydrogen sensor. JPhysChemC.

[21] Shi LC, Liu J, Puxian G, Zhang LY, Dragomir D, Tummala R, Wang LZ (2006). ZnO nanobelt/nanowire Schottky diodes formed by dielectrophoresis alignment across Au electrodes. Nano Lett.

[22] Zhang X, Jie J, Deng W, Shang Q, Wang J, Wang H, Chen X, Zhang X (2016). Alignment and patterning of ordered small-molecule organic semiconductor micro-/nanocrystals for device applications. Adv Mater.

[23] Law JBK, Thong JTL (2006). Simple fabrication of a ZnO nanowire photodetector with a fast photoresponse time. Appl Phys Lett.

[24] Jin Y, Wang J, Sun B, Blakesley JC, Greenham NC (2008). Solution-processed ultraviolet photodetectors based on colloidal ZnO nanoparticles. Nano Lett.

[25] Wu JJ, Liu SC (2002). Low-temperature growth of well-aligned ZnO nanorods by chemical vapor deposition. Adv Mater.

[26] Yang JL, An SJ, Park WI, Yi GC, Choi W (2004). Photocatalysis using ZnO thin films and nanoneedles grown by metal–organic chemical vapor deposition. Adv Mater.

[27] Wenas WW, Yamada A, Konagai M, Takahashi K (1991). Textured ZnO thin films for solar cells grown by metalorganic chemical vapor deposition. Jpn J Appl Phys.

[28] Kaidashev E, Lorenz MV, Von WH, Rahm A, Semmelhack HC, Han KH, Benndorf G, Bundesmann C, Hochmuth H, Grundmann M (2003). High electron mobility of epitaxial ZnO thin films on c-plane sapphire grown by multistep pulsed-laser deposition. Appl Phys Lett.

[29] Jin B, Im S, Lee SY (2000). Violet and UV luminescence emitted from ZnO thin films grown on sapphire by pulsed laser deposition. Thin Solid Films.

[30] Jeong SH, Kim BS, Lee BT (2003). Photoluminescence dependence of ZnO films grown on Si (100) by radio-frequency magnetron sputtering on the growth ambient. Appl Phys Lett.

[31] Puchert M, Timbrell P, Lamb R (1996). Postdeposition annealing of radio frequency magnetron sputtered ZnO films. J Vac Sci Technolo A: Vac Surf Films.

[32] Al AR, Atanas J, Ajaka M, Zaatar Y, Ferblantier G, Sauvajol J, Jabbour J, Juillaget S, Foucaran A (2005). Characterization and Raman investigations on high-quality ZnO thin films fabricated by reactive electron beam evaporation technique. J Cryst Growth.

[33] Mahmood A, Ahmed N, Raza Q, Khan TM, Mehmood M, Hassan M, Mahmood N (2010). Effect of thermal annealing on the structural and optical properties of ZnO thin films deposited by the reactive e-beam evaporation technique. Phys. Scripta.

[34] Chaturvedi N, Swami SK, Kumar A, Dutta V (2014). Role of ZnO nanostructured layer spray deposited under an electric field in stability of inverted organic solar cells. Sol Energy Mater Sol Cells.

[35] Kamalasanan M, Chandra S (1996). Sol-gel synthesis of ZnO thin films. Thin Solid Films.

[36] Gutruf P, Walia S, Sriram S, Bhaskaran M (2016). Visible-blind UV imaging with oxygen-deficient zinc oxide flexible devices. Adv Electron Mater.

[37] Hong K, Kim SH, Lee KH, Frisbie CD (2013). Organic electronics: printed, sub-2V ZnO electrolyte gated transistors and inverters on plastic. Adv Mater.

[38] Liang S, Sheng H, Liu Y, Huo Z, Lu Y, Shen H (2001). ZnO Schottky ultraviolet photodetectors. J Cryst Growth.

[39] Young SJ, Ji LW, Chang SJ, Su YK (2006). ZnO metal–semiconductor–metal ultraviolet sensors with various contact electrodes. J Cryst Growth.

[40] Liao X, Liao Q, Zhang Z, Yan X, Liang Q, Wang Q, Li M, Zhang Y (2016). A highly stretchable ZnO fiber-based multifunctional nanosensor for strain/temperature/UV detection. Adv Funct Mater.

[41] Yan SS, Zhang H, Azad F, Su SC (2017). Fabrication, structural characterization and optical properties of oversized ZnO microwires. Nano.

[42] Willander M, Yang L, Wadeasa A, Ali S, Asif M, Zhao Q, Nur O (2009). Zinc oxide nanowires: controlled low temperature growth and some electrochemical and optical nano-devices. J Mater Chem.

[43] Djurišić AB, Leung YH (2006). Optical properties of ZnO nanostructures. Small.

[44] Yang Y, Guo W, Qi J, Zhao J, Zhang Y (2010). Self-powered ultraviolet photodetector based on a single Sb-doped ZnO nanobelt. Appl Phys Lett.

[45] Wang X, Zhou J, Song J, Liu J, Xu N, Wang ZL (2006). Piezoelectric field effect transistor and nanoforce sensor based on a single ZnO nanowire. Nano Lett.

[46] Clark SJ, Segall MD, Pickard CJ, Hasnip PJ, Probert MI, Refson K, Payne MC (2005). First principles methods using CASTEP. Z. Krist-Cryst. Mater.

[47] Soler J, Artacho E, Gale J, Garcia A, Junquera J, Ordejon P, Sanchez-Portal D (2002) The SIESTA method for ab initio order-N materials simulation. J Phys Condens Mater 14(11):2745

[48] Perdew JP, Burke K, Ernzerhof M (1996). Generalized gradient approximation made simple. Phys Rev Lett.

[49] Xia JB (1996). Electronic structure of quantum spheres and quantum wires. J Lumin.

[50] Kind H, Yan H, Messer B, Law M, Yang P (2002). Nanowire ultraviolet photodetectors and optical switches. Adv Mater.

[51] Li Q, Liang Y, Wan Q, Wang T (2004). Oxygen sensing characteristics of individual ZnO nanowire transistors. Appl Phys Lett.

[52] Calarco R, Marso M, Richter T, Aykanat AI, Meijers R, Hart v, Stoica T, Lüth H (2005). Size-dependent photoconductivity in MBE-grown GaN-nanowires. Nano Lett.

